# Eight-channel high-speed electrical impedance tomography device implemented on a programmable system on a chip

**DOI:** 10.1016/j.ohx.2025.e00667

**Published:** 2025-07-09

**Authors:** Fausto Andrés Escobar, Carlos Felipe Rengifo, Víctor Hugo Mosquera

**Affiliations:** a Universidad Mariana, Street 18 No. 34-104, Pasto, Nariño, Colombia; b Universidad del Cauca, Department of Electronics, Instrumentation and Control, Street 5 No. 4-70 Popayán, Colombia

**Keywords:** Bioimpedance measurement, EIT system, EIT image reconstruction, PSoC, Global impedance

## Abstract

This study proposes an electrical impedance tomography (EIT) device based on a programmable system on a chip (PSoc). The EIT-PSoC system is implemented using two PSoC 5LP platforms. A resistive phantom is used to study frame frequency (fps), accuracy (Ac), and signal-to-noise ratio (SNR). A saline phantom, along with both conductive and non-conductive objects, is employed to evaluate the system’s ability to detect changes in impedance distribution. Finally, the dielectric characteristics of the human lower pelvis is emulated using four agar phantoms, allowing an evaluation of the EIT-PSoC system’s performance in response to changes in fluid volume and conductivity. Experiments conducted on the resistive phantom to characterize the EIT-PSoC system demonstrate a frame frequency of 100 fps, a median SNR of 63.59 dB, and an accuracy of 95.39% when using a 0.98 mA sinusoidal current signal at 50 kHz. EIT image reconstruction shows that the proposed system can distinguish impedance changes in the saline phantom. Additionally, by utilizing the global impedance (GI) index and the agar phantoms, the EIT-PSoC system can detect changes in volume and conductivity, making this system a promising alternative for monitoring the volume and conductivity of biological fluids.

## Graphical abstract

**Figure:**
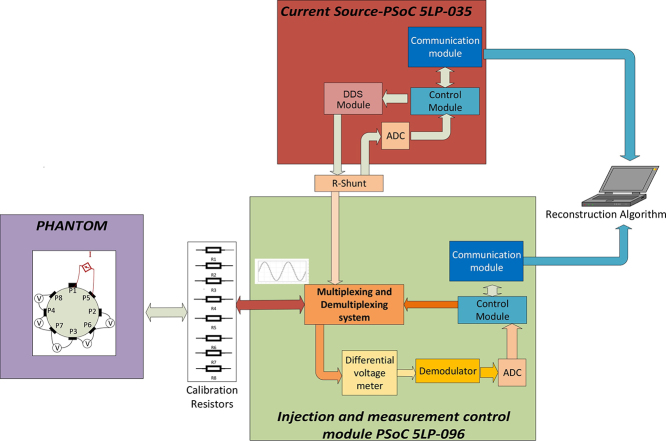

**Table:** 

**Specifications table**	

Hardware name	*Electrical impedance tomography system on programmable system on a chip (EIT-PSoC)*
Subject area	*Engineering and material science.*
Hardware type	*Measurement bioimpedance system.*
Closest commercial analog	*No commercial analog is available.*
Open source license	*CC BY 4.0.*
Cost of hardware	*245.95 USD.*
Source file repository	*https://data.mendeley.com/datasets/8cnn9znsrz/3*

	

## 1 Hardware in context

Electrical Impedance Tomography (EIT) is a non-invasive imaging technique that provides information about the internal conductivity distribution of a body or object. This technique involves injecting a low-level electrical current into the target area and measuring the resulting voltages on the body surface [1], [2]. To analyze the impedance changes, EIT generates images that depict the distribution of electrical properties within the examined region [3], [4].

The medical applications of EIT are based on the distinct electrical conductivities exhibited by biological tissues and fluids; when these are stimulated with an alternating electrical current, the resulting difference in impedance for each tissue or fluid can be measured using surface electrodes [5], [6]. The common EIT medical applications include lung imaging [7], [8], cerebral monitoring [9], [10], breast imaging [6], [11], and bladder monitoring [12], [13] showing promising results. EIT faces challenges such as limited spatial resolution, measurement noise, and sensitivity to electrode placement. The research in hardware and reconstruction algorithms aims to address these limitations [14], [15].

There are EIT systems based on Field Programmable Gate Arrays (FPGA), Digital Signal Processors (DSP), and microcontrollers. These EIT systems have a defined structure comprising: (i) a sinusoidal signal generation module, usually implemented by a Direct Digital Synthesizer (DDS); (ii) a voltage-controlled current source (VCCS); (iii) a multiplexing module for signal injection and measurement; (iv) a demodulation module; and finally, (v) a communication module [5], [7], [16], [17], [18], [19]. The usage of FPGA, DSP, and, microcontrollers depends on the temporal resolution required. For example, prototypes based on FPGA and DSP present a temporal resolution above 50 frames per second (fps), which allows the monitoring of biological processes with high temporal variability, such as the measurement of blood pressure [20], [21].

The EIT systems based on microcontrollers have a low frame rate, which allows the exploration of biological processes with low temporal variability, such as monitoring of bladder emptying and detection of cranial hemorrhages [13], [22]. On the other hand, spatial resolution is very important in image reconstruction and it is related with the number of electrodes. The greater the number of electrodes the best is the image reconstruction [23]; the increase in the spatial resolution implicates EIT systems with high speed of processing signals.

The detection limit of an EIT system is determined by its signal-to-noise ratio (SNR), which defines the minimum signal that can be reliably detected given the measurement noise [24]. SNR is thus an essential feature in EIT system design. On the other hand, sensitivity in EIT indicates the ability to detect changes in conductivity within the region of interest, maximizing the sensitivity of impedance measurements requires defining the measurement frequency that generates a significant change in voltage on electrodes due to small changes in impedance within the object under study [24]. Research results consistently show that using a 50kHz signal in an EIT system produces good sensitivity [9], [12]. Therefore, the proposed system generates a 50kHz alternating current signal.

EIT also allows the detection of organ size changes through the global impedance index (GI), which has been used for monitoring the bladder volume change [25], [26]; likewise, allows the detection of pulmonary conditions associated with an inhomogeneous tidal volume in the distribution of pulmonary ventilation, such as acute pneumothorax, respiratory distress syndrome (ARDS), and asthmatic bronchitis [27], [28] with goods results. GI estimates the volume of an organ or biological tissue due to the change in impedance distribution through the EIT image reconstruction matrix. Due to the potential of this technique for monitoring biological tissues, it will be taken into account to evaluate the system proposed in this work.

## 2 Hardware description

**Fig. 1:**
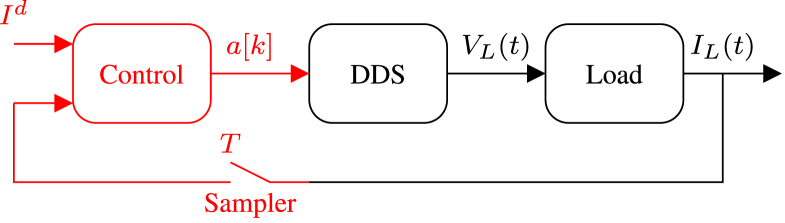
Closed control loop used to control the amplitude of the current through the object under study. The elements in red are discrete-time systems and signals, while the elements in black are continuous time.

Most EIT systems consist of a direct digital synthesizer (DDS), responsible for generating a sinusoidal voltage signal, which is sent into a voltage-controlled current source (VCCS). However, here we use a different approach previously proposed [29]. Instead of a VCCS, the amplitude of the voltage generated by the DDS is updated periodically as a function of the current through the load. Fig. 1 shows a closed control loop, where the voltage V(t) is the manipulated variable, the current $I_{L}\left(t\right)$ is the controlled variable, and $I^{d}$ is the desired current amplitude through the load. The controller samples the amplitude of the current $I_{L}\left(t\right)$ once per period to generate a discrete time signal named $I_{L}\left[k\right]$ and defined as the sequence $\{I_{L}\left(0\right)$, $I_{L}\left(T\right)$, $I_{L}\left(2\;T\right)$, …}. This signal and the desired current amplitude $I^{d}$ are used to calculate a[k], which is the amplitude of V(t) for kT≤t<(k+1)T. The amplitude a[k] is updated as follows [29]:

(1) $$a\left[k\right]=a\left[k-1\right]\;\frac{I^{d}}{I_{L}\left[k\right]},\;a\left[0\right]=1\;V$$

Using the same convention as for $I_{L}\left[k\right]$, V[k] is defined as the sequence {V(0), V(T), V(2T), …}. When a[k] is a voltage amplitude that can be generated by the DDS, then V[k] is equal to a[k] (Fig. 1)

$$V\left[k\right]=V\left[k-1\right]\;\frac{I^{d}}{I_{L}\left[k\right]},\;V\left[0\right]=1\;V$$

The above controller does not require any knowledge of the resistance load $R_{L}$. It depends only on the previous voltage amplitude V[k−1], the actual amplitude of the current through the load $I_{L}\left[k\right]$, and on the desired current amplitude $I^{d}$. For the first period (k=0), the value of $I_{L}\left[0\right]$ results from the Ohm’s Law

$$I_{L}\left[0\right]=\frac{V\left[0\right]}{R_{L}}$$

Therefore

$$V_{L}\left[1\right]=V_{L}\left[0\right]\;\frac{I^{d}}{I_{L}\left[0\right]}=V_{L}\left[0\right]\;\frac{I^{d}\;R_{L}}{V_{L}\left[0\right]}=I^{d}\;R_{L}$$

For k=1,

$$I_{L}\left[1\right]=\frac{V_{L}\left[1\right]}{R_{L}}=\frac{I^{d}\;R_{L}}{R_{L}}=I^{d}$$

The last equation shows that for the second period of the signal (k=1), the amplitude of the current through the load is equal to the desired current $I^{d}$, providing that $V_{L}\left[1\right]$ is a voltage amplitude belonging to the range of voltages of the DDS.

**Fig. 2:**
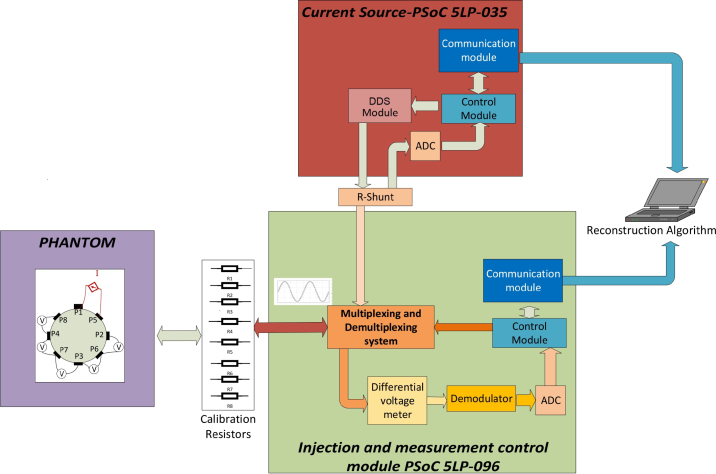
Scheme of EIT-PSoC system proposed.

The DDS generates a signal with frequencies ranging from 2Hz to 50kHz, which is directed to a multiplexer. The multiplexer applies this signal as a voltage to the object under study through a designated pair of consecutive electrodes. Meanwhile, the root-mean-square (RMS) of the differential voltages between each remaining electrode pair (those not used to apply the initial voltage) is measured through a differential amplification stage. Finally, the voltages are transmitted to a computer, where the image of the impedance distribution is reconstructed [30], [31]. The architecture of the proposed EIT system is introduced in Fig. 2.

Cypress’s PSoC devices integrate configurable analog and digital peripherals, along with memory and a single-chip microcontroller, making them attractive for highly integrated hybrid electronics applications. The CY8C58LP family is the one with the best features, it has a 32-bit ARM Cortex-M3 Microcontrollers with speeds of up to 80MHz and 1.25 DMIPS/MHz CPU and direct memory access (DMA). It also has programmable digital and analog peripherals with flexible routing, 256kB of program memory and 64kB of variable memory. The CY8C58LP family integrates a maximum of 100 analog and digital functions. Regarding the analog peripherals, the PSoC 5LP includes: one delta-sigma analog-to-digital converter (ADC) configurable from 8 to 20 bits, two ADC 12-bit SAR type, four 8-bit DAC, four analog comparators, four op-amps, four blocks of continuous-time switched capacitor (SC/CT) to create Programmable Gain Amplifiers (PGA), transimpedance Amplifiers (TIA), mixers, sample and hold circuits. Considering the features of the CY8C58LP, the prototype proposed in this work is composed of two of these devices. One for implementing the DDS, VCCS, and ADC; and the other for multiplexing and demultiplexing modules, demodulation, and communication (Fig. 2). Additionally, the electrodes and ports of the PSoC 5LP-086 are connected via 100 Ω resistors in order to: (i) match the impedance between the electrodes and the PSoC inputs, (ii) protect against voltage spikes, (iii) maintain defined logic levels in the absence of a signal, and (iv) match signal levels to the PSoC ADC input range and assist in linearizing the sensor signal. The development of each module of the proposed EIT prototype is detailed below.

### 2.1 Direct digital synthesizer

**Fig. 3:**
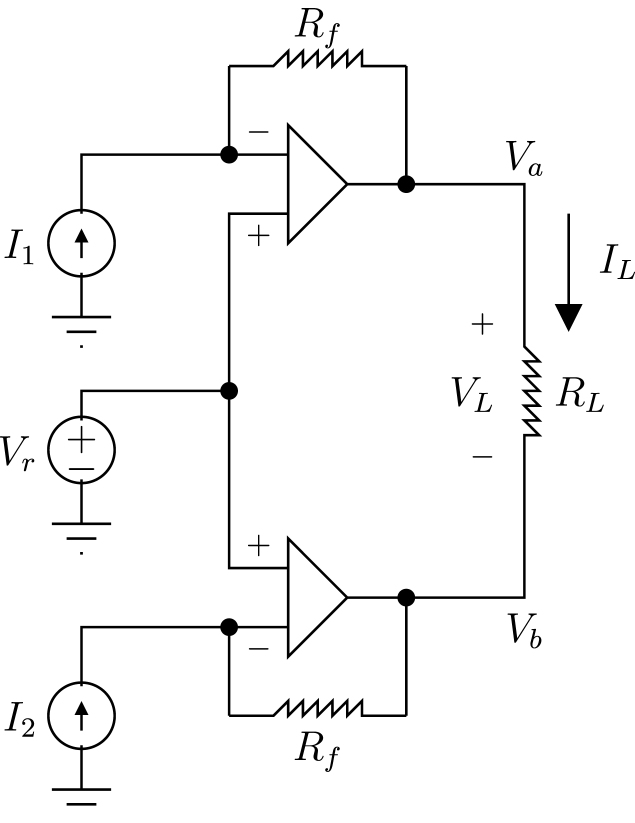
Sinusoidal signal generator based on PSOC IDACs [29].

The DDS, presented in Fig. 3, uses two Current Digital to Analog Converters (IDAC) in source mode to generate positive currents, which are converted in to voltages using trans-impedance amplifiers (TIAs), and then these voltages are subtracted to obtain a bipolar signal. The model of the DDS is obtained as follows:

• *Model for the upper amplifier*. Due to the high impedance of the amplifier the current $I_{1}$ is equal to $I_{f1}$. Therefore:
  $$I_{1}=\frac{V_{r}-V_{a}}{R_{f}}$$
  Solving for $V_{a}$
  (2) $$V_{a}=V_{r}-R_{f}\;I_{1}$$
• *Model for the lower amplifier*. Using the same procedure as for the upper amplifier, we obtain: 33
  $$I_{2}=\frac{V_{r}-V_{b}}{R_{f}}$$
  Solving for $V_{b}$
  (3) $$V_{b}=V_{r}-R_{f}\;I_{2}$$
• *Voltage across the load*. This is the difference between the voltages $V_{a}$ and $V_{b}$
  (4) $$V_{L}=V_{a}-V_{b}=\left(V_{r}-R_{f}\;I_{1}\right)-\left(V_{r}-R_{f}\;I_{2}\right)=R_{f}\left(I_{2}-I_{1}\right)$$

**Fig. 4:**
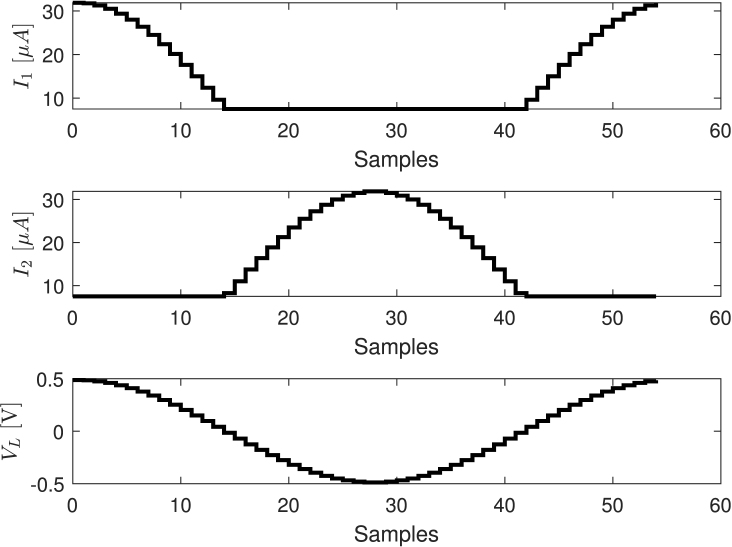
Currents generated by the IDACs and the voltage $V_{L}$ when $R_{f}=20\;k\Omega$.

Despite $V_{L}$ does not depends on $V_{r}$, this last value must be carefully calculated to ensure $V_{a}>0$ and $V_{b}>0$. This requirement is due to the fact that the PSOC family only generates positive voltages. Therefore, according to Eqs. (2), (3), $V_{r}$ should satisfy the following inequalities: $V_{r}>R_{f}\;I_{1}$ and $V_{r}>R_{f}\;I_{2}$. Both IDACs were configured to generate currents between 0 and 31.875μA when the 8-bit IDAC register varies from 0 to 255. The 8-bit IDAC register was updated 55 times per period of the cosine signal, whose frequency was 50kHz. IDAC’s register are updated by Direct Memory Access (DMA) using the following values:

(5) $$\left(\begin{array}{ccc} S_{i} & = & \cos\left(\frac{2\;i\;\pi }{55}\right)\\ D_{i}^{+} & = & \left\lfloor \frac{255\;V_{L}\left[k\right]}{31.875\cdot 10^{-6}\;R_{f}}\max\left(0,+S_{i}\right)\right\rfloor \\ D_{i}^{-} & = & \left\lfloor \frac{255\;V_{L}\left[k\right]}{31.875\cdot 10^{-6}\;R_{f}}\max\left(0,-S_{i}\right)\right\rfloor \end{array}\right\}\;i=0,1,2,\ldots ,54$$

where ⌊x⌋ is the round operator, $D_{i}^{+}$ and $D_{i}^{-}$ are the integer values set in the 8-bit IDACs registers for the sample i of the period k. The Fig. 4 presents the currents generated by the IDACs $I_{1}$ and $I_{2}$, as well as the voltage $V_{L}$ when $R_{f}=20\;k\Omega$. To minimize distortion caused by IDAC’s commutation, they are used in source mode, and their output current is converted into voltage through a trans-impedance amplifier (TIA); as detailed in [29].

Small values of V[k] lead to current signals spanning along the least significant bits of the IDAC register, which implies distortion in the generated cosine signal due to quantization. For example, when $R_{f}=20\;k\Omega$ and V[k]<0.16V, the registers $D_{i}^{+}$ and $D_{i}^{-}$ varies between 0 and 63. This means that only five of the eight bits of the register are used. To avoid this problem, $R_{f}$ is dynamically varied depending on V[k] according to the ranges indicated in Table 1

The previous design could have been simplified using one IDAC in source mode for the positive semicycle of the cosine signal and another in sink mode for the negative semicycle. However, this arrangement generated distortion from frequencies above 500Hz. The implementation of DDS was done in the PSoC Creator 4.0 development environment, for a PSoC 5LP CY8C5868AXI-LP035 device, the design of a DDS is detailed in [29].

**Table 1:** Values of $R_{f}$ as a function of the desired output voltage.

Lower V[k]	Upper V[k]	$R_{f}\;k\Omega$	Lower $D_{i}^{+}/D_{i}^{-}$	Upper $D_{i}^{+}/D_{i}^{-}$
0.15000	0.63750	20	60	255
0.63750	0.95625	30	170	255
0.95625	1.27500	40	191	255
1.27500	2.55000	80	127	255
2.55000	3.82500	120	170	255
3.82500	4.62500	250	122	148

### 2.2 Differential Voltage Meter (DVM)

**Fig. 5:**
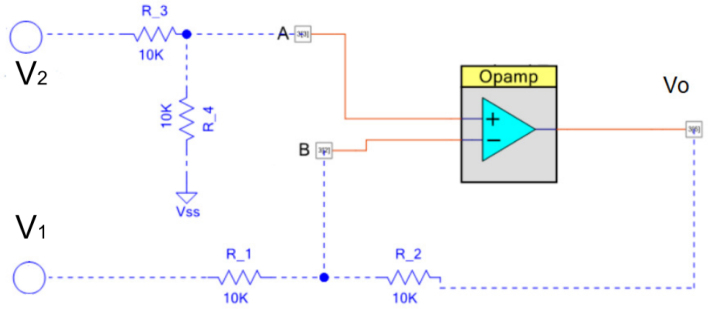
OP-Amp differential.

The DVM measures the electrical potential between electrodes connected to the inputs $V_{1}$ y $V_{2}$. The controlled-gain differential mode operational amplifier (Fig. 5), amplifies the difference between the voltage inputs. The advantage of the differential amplifier is that it rejects common mode noise.

Eq. (6) presents the model of the DVM when $R_{1}=R_{2}=R_{3}=R4=10\;k\Omega$

(6) $$V_{0}=V_{2}-V_{1}$$

### 2.3 Current measurement

The current is measured indirectly by recording the voltage across a 100Ω resistor placed in series with the load impedance $R_{L}$. Using Ohm’s law, the current flowing through both the shunt resistor and the load is calculated based on the measured voltage. The voltage-analog-to-digital converters (VADCs) of the PSoC have a significantly lower sampling frequency compared to the IDACs. As a result, achieving fine control over the amplitude and shape of the sinusoidal output current is not feasible. To address this limitation, a minimalist control approach was adopted to optimize the use of hardware resources. Specifically, a configuration was implemented that controls the peak amplitude of the current signal by reading the voltage across the shunt resistor at the moment when the output signal reaches its maximum amplitude. The VADC of successive approximations (SAR) in differential mode with sampling frequency of 1.125 Mps and 10-bit resolution uses an end-of-conversion interruption to deliver the measured voltage between the terminals of the shunt resistance, to the control algorithm.

### 2.4 Injection pattern

**Fig. 6:**
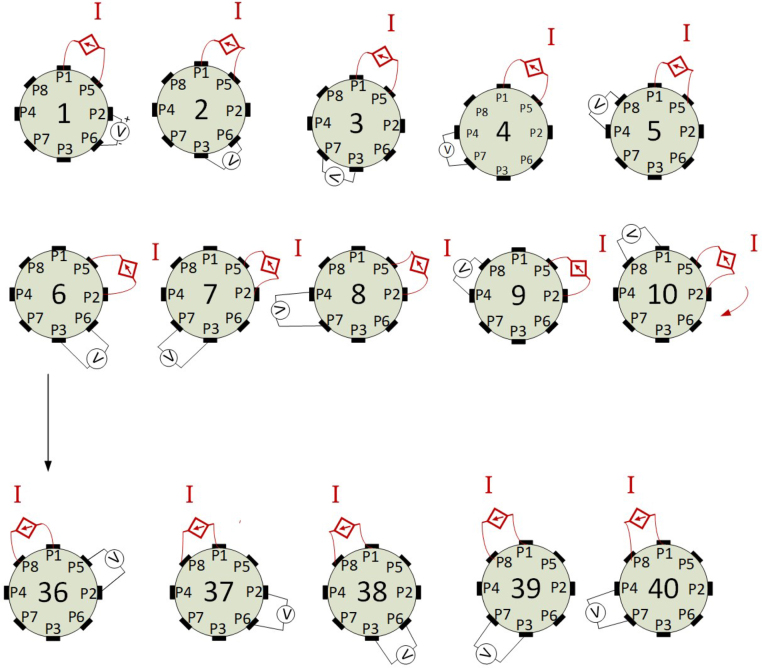
Adjacent measurement and injection patterns.

The implemented injection and measurement pattern is adjacent (Fig. 6), where P1 to P8 refer to the pins connected to the phantom. The injection sequence begins with current injection through pins P1-P5 and voltage measurements across the pairs: P2-P6, P6-P3, P3-P7, P7-P4, and P4-P8. Subsequently, the injection is performed through P5-P2, and measurements are taken across the pairs: P6-P3, P3-P7, P7-P4, P4-P4, P4-P8, and P8-P1 forming a vector of five measurements as previously described. The process is completed when the pair P8-P1 is used for injection. The injection is maintained for a fixed period by the timer while the five voltage measurements are carried out. This procedure generates an 8 × 5 matrix, which is referred as a frame.

### 2.5 Multiplexing

**Fig. 7:**
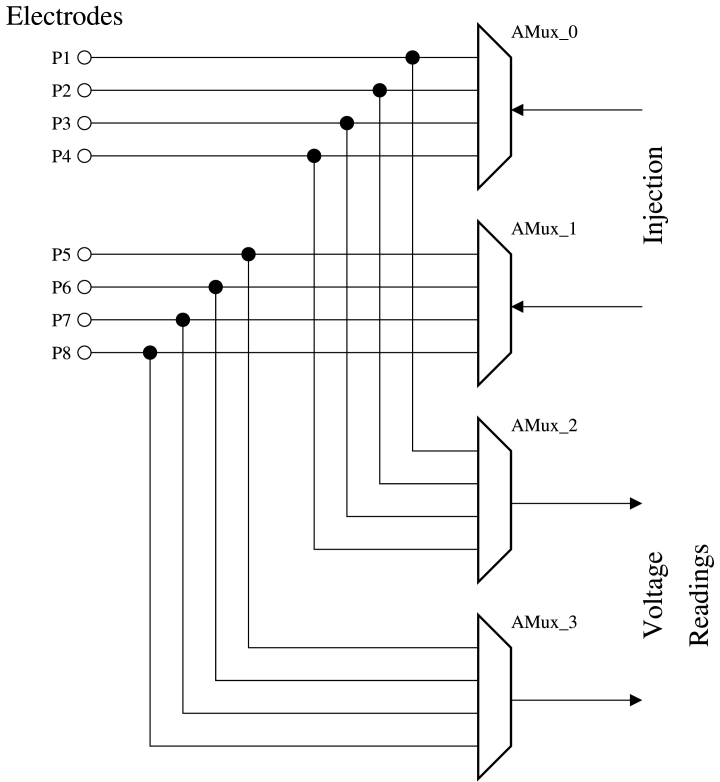
Multiplexing scheme.

The proposed EIT system uses four multiplexers (AMux0, AMux1, AMux2, AMux3) to inject current through two electrodes and read voltage across the remaining electrodes. AMux0 and AMux1 multiplex the current injection source, and the voltage measurements are demultiplexed by AMux2 and AMux3 (Fig. 7). The timing for the injection and measurement sequences are carried out by two interruptions using timers. One is in charge of the injection source through two electrodes of the phantom. While the other one is in charge of activating two channels for voltage reading. The complete scheme of the EIT-PSoC system can be seen in the folder EIT-PSoC Design of the repository https://data.mendeley.com/datasets/8cnn9znsrz/3.

### 2.6 Volume estimation with EIT

Global impedance (GI) has been applied to estimate bladder volume, presenting a linear correlation. The reconstruction matrix ($R^{f}$) of the EIT image is used for comparing the change of the impedance distribution of a partially filled bladder and an empty bladder and estimating the volume [13], [25], [26], [32]. The variations in the potential for any frame *f* are calculated using the following expression:

(7) $$\Delta v^{f}\left(k\right)=\frac{v_{nh}^{f}\left(k\right)-v_{h}\left(k\right)}{v_{h}\left(k\right)},k=1,\ldots ,N$$

where $\Delta v^{f}\left(k\right)$, $v_{nh}^{f}\left(k\right)$, and $v_{h}\left(k\right)$, are the kth elements of the vectors $Deltav^{f}$, $v_{nh}^{f}$, and $v_{h}$, where $v_{h}$ is the reference frame (homogeneous) and $v_{nh}^{f}$ are the measurements of inhomogeneous vectors; each frame has a size from 1 to N, where N corresponds to the number of independent measurements per frame, and in turn is the number of elements of $v_{h}$ and $v_{nh}^{f}$. subsequently, the pixel conductivity vector ($I_{f}=R^{f}\Delta v^{f}$) is calculated [25]. The sum of elements of $I_{f}$ for each frame is defined as the GI (Eq. (8)):

(8) $$GI=\overset{N_{f}}{\underset{f=1}{\sum }}\overset{N}{\underset{k=1}{\sum }}I^{f}\left(k\right)$$

Due to the promising results of ${\left|GI\right|}^{-1}$ in the volume monitoring of bladder [25], and study of respiratory system [27], [28]; this approach will be used for evaluating the performance of the proposed EIT system.

## 3 Design files summary

The figures of EIT-PSoC system design files are located in Table 2. On the other hand, in the repository (https://data.mendeley.com/datasets/8cnn9znsrz/3) are located the next folders of EIT-PSoC system:

• EIT-PSoC Design: The EIT-PSoC system PCB design files are located.
• PSoC programming: This folder contains the files to program the PSoC 5LP-096, and PSoC 5LP-035. The subfolder **VCCS-PSoC 5LP-035** has the files to implement the VCCS. The files for the demodulator and multiplexing are in the subfolder **Multiplexing and Demodulator-PSoC 5LP-096**.
• Image reconstruction: In this folder are the Matlab scripts that allow the reconstruction of EIT images employing the measures taken by EIT-PSoC system.
• Frames measurements: The Matlab scripts for capturing and storing the frames measured by the EIT-PSoC system are found in this folder.
• Frames Agar: In this folder are stored the measures obtained for calculating the SNR, and tests on phantom of agar

**Table 2:** Design file of EIT-PSoC.

Design filename	File type	Open source license	Location of the file
General scheme of EIT-PSoC system	Figure PNG	CC BY 4.0	Included in the paper (Fig. 1)
PCB of EIT-PSoC system	Figure PNG	CC BY 4.0	Included in the paper (Fig. 8)
Scheme of voltage controlled current source in PSoC editor	Figure PNG	CC BY 4.0	Included in the paper (Fig. 2)
Differential voltage meter	Figure PNG	CC BY 4.0	Included in the paper (Fig. 3)
Amplitude Demodulator	Figure PNG	CC BY 4.0	Included in the paper (Fig. 4)
Multiplexing	Figure PNG	CC BY 4.0	Included in the paper (Fig. 5)
Schematic of EIT-PSoC	Figure PNG	CC BY 4.0	Included in the paper (Fig. 7)

## 4 Bill of materials summary

The list of materials used in the design of the EIT-PSoC system is presented in Table 3.

**Table 3:** Bill of materials of the EIT-PSoC.

Designator	Component	Number	Cost per unit-currency	Total cost-currency	Source of materials	Material type
PSoC 5LP Development Kit (CY8C5868AXI -LP035 100 TQFP)	Programmable SoC, combining high-precision analog and digital peripherals	1	115.11	115.11	Mouser.com	Electronic

PSoC 5LP Development Kit (CY8C5888AXI -LP096 100-TQFP)	Programmable SoC, combining high-precision analog and digital peripherals	1	111.09	111.09	Utmel.com	Electronic

Bluetooth communication module	1	1.39	1.39	Amazon.com	Electronic	

Battery lithium 9V	Battery	1	18.36	18.36	Amazon.com	Other

**Total**				**245.95**		

## 5 Build instructions

The programming IDE used during the development of the work is PSoC Creator 4.2, which can be downloaded from the Cypress website: https://www.cypress.com/products/psoc-creator-integrated-design-environment-ide. The programming of the PSoC’s, follows the following procedure:

### 5.1 VCCS current source (PSoC 5LP-035)

• Open the project called *EIT-PSoC*, located in the VCCS-PSoC 5LP-035 folder.
• Select the Pins tab and verify that the card series CY8C5868AXI-LP035 100 TQFP matches, and the connection assignment.
• Once the environment configuration has been verified, select the “Clean and Build” option to compile the project.
• Finally, connect the PSoC 5LP-035 card to the USB port of the PC, and in the Debug tab select the “Program” option, which Will download the program to the card.

### 5.2 Multiplexing and demodulator (PSoC 5LP-096)

• Open the Project called *Measurement*, located in the Multiplexing and Demodulator-PSoC 5LP-096 folder.
• Select the Pins tab and verify that the card series CY8C5888AXI-LP096 100-TQFP matches, and the connection assignment.
• Once the environment configuration has been verified, select the “Clean and Build” option, to compile the project, and connect the PSoC 5LP-096 card to the USB port of the PC, and in the Debug tab select the “Program” option. Which will download the program to the card, just like in the previous Project.

## 6 Operation instructions

• Once the electrodes of the EIT-PSoC system are connected to the resistive, saline, or agar phantom, both PSoCs are polarized with 5 V.
• The injection and measurement process starts with executing the Matlab script **Capture.m**. The measurements are separated by 8 × 5 frames and stored in a text file in the CaptureData folder, using the **Splitvectors.m**, **Frame.m**, and **Save_Frame.m** functions included in **Capture.m**. These scripts can be found in the Frames-Measurements folder.
• The SNR is calculated by running the **SNR.m** script, located in the SNR folder. Previously, The measurements of resistive phantom, obtained by following the instructions in the previous point, must be stored in the Frames_PSoC folder of the SNR folder.
• For the image reconstruction and volume estimation, download EIDORS (https://eidors3d.sourceforge.net/) and run the **stardup.m** script from Matlab, which script is located in the folder where is downloads EIDORS.
• For EIT image reconstruction (found in the Image_Reconstruction folder), the captured frames are copied into the Frames_PSoC folder. Ensure that Frame0.txt contains the homogeneous measurements (phantom with saline only or agar phantom without cavities), while the other frames correspond to the non-homogeneous measurements (phantoms with tubes inside or cavities filled with saline solution). Then, execute the **EIT_Image_Reconstruction.m** script.
• The GI is calculated by executing the *GI_Index.m* script located in folder GI. The measurements taken by EIT−PSoC system must be copied to the Frames_PSoC folder, assuring the first frame is the homogeneous measurement and the other frames would be the non-homogeneous measurement.

## 7 Validation and characterization

The characteristics of the EIT-PSoC system, such as the signal generated by the DDS, and injection current, were evaluated with resistive loads; the SNR, switching times, and measurement accuracy, were analyzed through experiments with a resistive phantom. On the other hand, the performance of the EIT image reconstruction was evaluated using a phantom with saline solution. Finally, with four agar phantoms, which emulate the dielectric characteristics of the human lower pelvis and urine conductivity [25], were used to study the sensibility to conductivity changes and volume. The experiments were carried out in controlled environment, with 21°C temperature and humidity of 65%. The details of experiments are explained below.

### 7.1 Performance of VCSS and demodulator modules

The first experiment uses a 1kΩ resistor connected in series with the shunt resistor as a load. By varying the amplitude of the signal generated for the DDS between 24.5, 49, 98, and 196 mV (peak-to-peak) at a frequency of 50kHz, the voltage across the resistive loads is measured by the implemented demodulator. For the analysis, 200 measurements of the electric potential on the load were taken. The measurements of the demodulator are compared with the measurements carryout by the BK PRECISION 2540B oscilloscope. The results of this experiment are shown in Table 4. The relative and absolute errors, less than 0.43%, and 0.02 respectively, show that the demodulator performs best when the DDS generates a voltage of 98mV, indicating coherence and consistency between measurements. For subsequent experiments, the voltage generated by the DDS will be set to 98mV.

In a second experiment, the current of the implemented VCCS is evaluated. The DDS signal is set to 98mV for frequencies of 1kHz, 10kHz, and 50kHz, and the resistive load takes values of 10Ω, 100Ω, and 1000Ω. Table 5 shows that the EIT system generated a current signal of 0.98mA with a standard deviation of 0.01mA, independent of load and frequency. Furthermore, the RMS voltage shows little variation in the resistive loads. These results demonstrate the high performance of the prototype implemented on PSoC.

**Table 4:** Absolute, relative error and measurement accuracy of the modulator implemented on PSoC on resistive load.

Voltage (mV)	Oscilloscope measurement (V)	Demodulator measurement (V)	RE (%)	AE (mV)
24.5	1.35	1.24	8.14	0.11
49	2.54	2.39	5.90	0.15
**98**	**4.60**	**4.58**	**0.43**	**0.02**
196	6.99	6.95	0.57	0.04

**Table 5:** Results of current generated by the VCCS for different resistive loads.

Frequency (kHz)	RMS demodulator measurement (mV)		
	R = 10 Ω	R = 100 Ω	R = 1000 Ω
1	6.95	68.56	694.22
10	6.97	69.20	692.13
50	6.98	69.17	694.70
**Current (mA)**	**0.98 ± 0.01**	**0.97 ± 0.01**	**0.98 ± 0.01**

#### 7.1.1 SNR and accuracy of the EIT-PSoC system

**Fig. 8:**
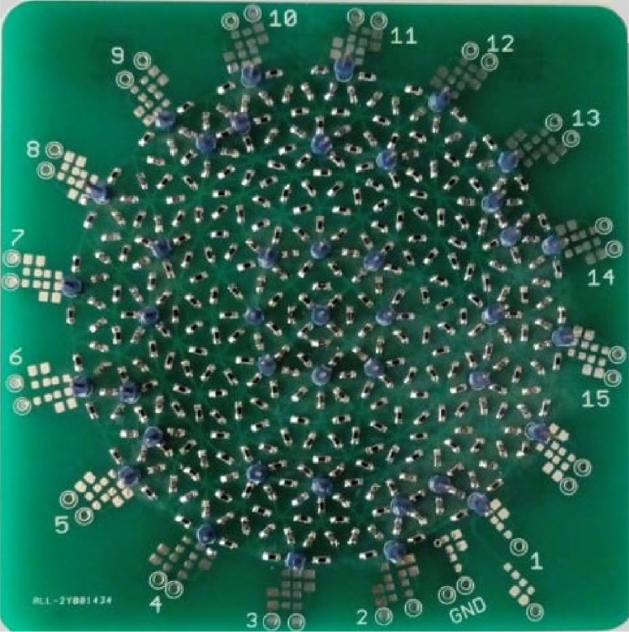
Resistive phantom.

To determine, the SNR and the accuracy of the proposed EIT-PSoC system, a 2D resistive phantom (Cardiff EIT phantom) is used (Fig. 8). The procedure starts by connecting the 8 electrodes with an adjacent configuration (Fig. 6). Subsequently, the magnitude of the current signal is fixed at 0.98mA. The SNR is calculated for different frequencies (1kHz, 10kHz, and 50kHz,) and switching times of the measurement AMUX (1ms, 100ms, and 1000ms). For the analysis, 48 frames are stored for each frequency and switching time. The obtained frames allow establishing the SNR and accuracy (Ac) through Eqs. (9), (10) respectively.

(9) $$SNR_{i}=20log\left(\frac{\left|mean\left(m_{\left(i,j\right)}\right)\right|}{\sqrt{Var\left(m_{\left(i,j\right)}\right)}}\right)$$

(10) $$Ac_{i}=\left(1-\left|\frac{mean\left(m_{\left(i,j\right)}\right)-m_{\left(i,j\right)}^{T}}{m_{\left(i,j\right)}^{T}}\right|\right)\times 100$$

where $m_{\left(i,j\right)}$ represents the ith measure, $mean\left[m_{\left(i,j\right)}\right]$, and Var[m(i,j)] are average and variance of $m_{\left(i,j\right)}$ respectively.

The SNR and accuracy results are presented in Table 6, These indicate that the switching time of 1ms for voltage measurements presents the best characteristics (mean SNR of 63.59 dB and 95.39% accuracy) using an injection current of 0.98mA at a frequency of 50kHz. When the switching time is increased to 10 and 100 ms, transients appear that directly affect the quantization process, causing a detriment to SNR and accuracy. This is because when switching a new channel, there is a transient that adds dispersion to the measurement; the dispersion improves if we wait for 1ms to measurement; but if the delay is greater than 1ms, the dispersion grows, due to a slow drift in the measured voltages that can be observed at the signal inputs. Therefore, the measurement time of 1ms at a frequency of 50kHz will be used for the subsequent experiments in the phantom because it generates the best performance of the proposed EIT system.

**Table 6:** SNR and accuracy on resistive phantom 2D.

kHz	Accuracy (%)			SNR (dB)		
	Max	Mean	Min	Max	Mean	Min
Switching time of 1 ms						

1	99.97	98.46	96.21	63.80	51.04	46.05
10	99.89	98.89	95.96	61.23	60.50	48.89
50	98.58	**95.39**	92.89	65.83	**63.59**	48.51

Switching time of 10 ms						

1	99.14	95.50	92.46	60.03	50.65	46.75
10	78.32	76.39	74.52	83.08	54.68	55.46
50	88.82	85.39	82.59	54.33	53.59	46.19

Switching time of 100 ms						

1	79.95	76.94	71.53	12.33	9.80	8.05
10	79.82	76.53	66.45	12.52	10.02	6.91
50	79.86	73.09	64.98	14.27	11.46	8.48

### 7.2 Image reconstruction and volume estimation

**Fig. 9:**
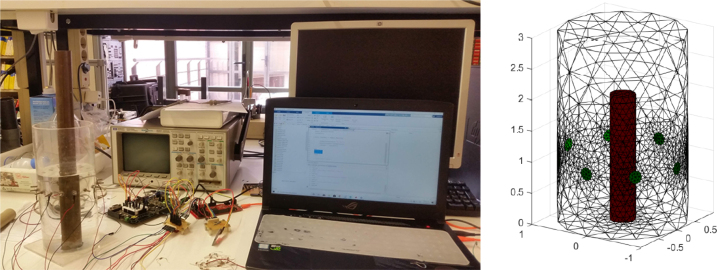
Experiment setup with Saline phantom (left), and FEM model (right).

The performance of the EIT system in image reconstruction is evaluated using a tank filled with a saline solution with a radius of 7cm and a height of 30cm. The conductivity of the saline solution used in the experiments is 0.278 S/m, measured with a Milwaukee MW802 Meter. During the image reconstruction process, a polyvinyl chloride cylinder with a radius of 2.1cm and a copper cylinder with a radius of 2.15cm are introduced into the tank independently and jointly. Fifty frames are stored for each experiment. Finally, the frames are processed using an application in Matlab-EIDORS to generate images of impedance distribution. The experiment setup is shown in Fig. 9.

**Fig. 10:**
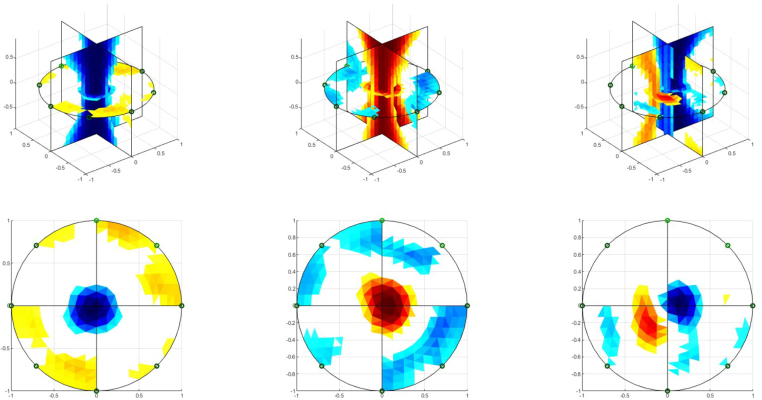
EIT image reconstruction of the saline phantom. 3D conductivity distribution (up) and 2D conductivity distribution in the arrangement of electrodes plane (down).

The EIT image reconstruction is carried out by using the conductivity distribution in the tank with saline solution (homogeneous measurements) and the tank with the conductive and non-conductive objects (inhomogeneous measurements) as references. The application MATLAB-EIDORS reconstructs the conductivity distribution using a finite element model (FEM) of 25 511 elements (Fig. 9). The EIT image reconstruction algorithm used is the Gauss–Newton method in conjunction with the Total Variation regularization algorithm, these are commonly employed in EIT medical applications [30], [31]. Fig. 10 shows the 2D and 3D conductivity distribution when conductive and non-conductive objects are introduced individually and simultaneously into phantom (non-conductive bar (left), conductive bar (center), and non-conductive and conductive bars (right)). The EIT images reveal the conductivity changes inside the phantom, distinguishing between low and high conductivity objects both individually, and together. These results demonstrate the ability of the developed EIT system to detect different conductivity changes in the study region.

**Fig. 11:**
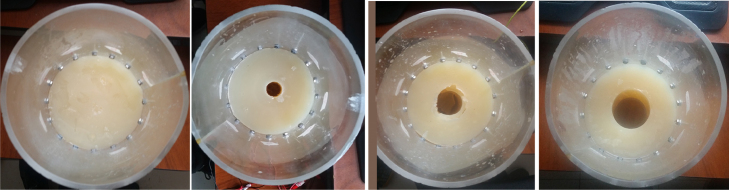
Agar phantoms.

Four agar phantoms were used in the experiments to study the performance of the EIT-PSoC system in terms of simultaneous changes in volume and conductivity. In a cylindrical acrylic tank with a radius of 7cm and a height of 30cm, the electrical characteristics of the lower pelvis were emulated. The lower pelvis tissue was emulated using 20g of biological LB agar (LENNOX) diluted in a liter of saline solution with 0.193S/m conductivity; generating a conductivity of 0.217S/m [25]. A phantom without a cavity was used for homogeneous measurements, while the other three phantoms had cavities with radii of 2.7cm, 2.5cm, and 1.3cm to emulate volume changes. The cavities were filled with saline solutions with conductivity of 1.227S/m, 1.890S/m, and 2.07S/m to simulate the variation in the conductivity of biological fluids. The conductivities of the phantoms and saline solutions were measured with a CRISON GLP-32 conductivity analyzer. Fig. 11 shows the agar phantoms.

For volume estimation on the agar phantom, the global impedance (GI) method is used (Section 2.5), with an 8-electrode ring and injection and measurement adjacent pattern. For each frame, the reconstruction matrix ($R^{f}$) is calculated from a 2D-TIE image of 32 × 32 pixels, $R^{f}$ has 1024 rows and 40 columns for a FEM cylindrical of 25 511 elements.

**Fig. 12:**
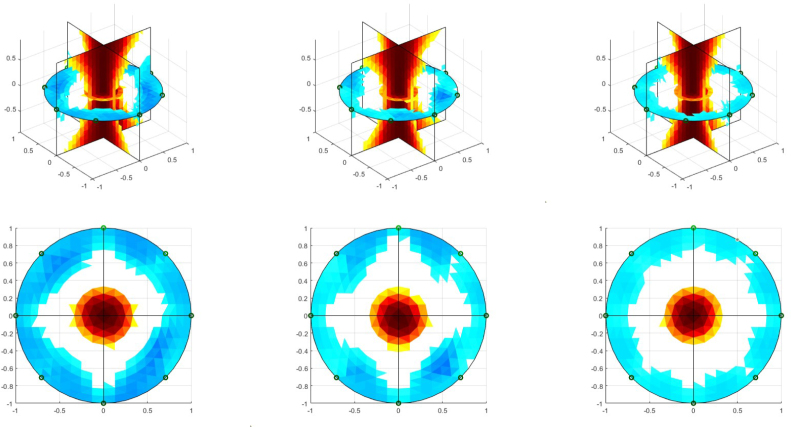
EIT image reconstruction 3D of the agar phantom (up) and the conductivity distribution in the plane of electrodes (down). Left: large cavity. Center: medium cavity. Right: small cavity.

The results on agar phantoms are presented in Fig. 12. The 3D impedance distribution evidences the difference between the conductivity of the saline solution in the cavity and the background. On the other hand, the EIT image reconstruction of the electrode plane shows the difference between the radii of the large and medium cavities (2.7cm and 2.2cm, respectively). However, the EIT image for the small cavity (radius of 1.3cm) does not distinguish its size difference from the phantom with the medium cavity. These results indicate that the system designed with 8 electrodes and the Gauss–Newton algorithm has performs poorly when the object’s volume under study is small.

**Fig. 13:**
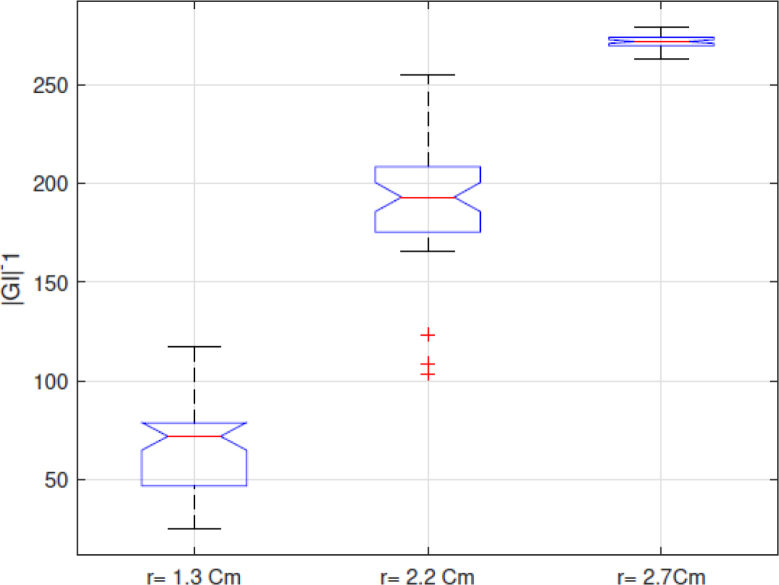
${\left|GI\right|}^{-1}$ for conductivity of 1.227S/m.

**Fig. 14:**
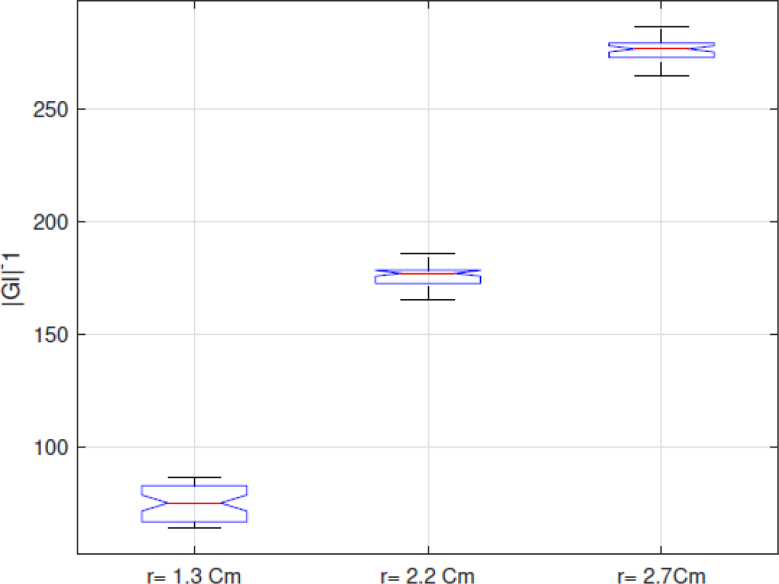
${\left|GI\right|}^{-1}$ for conductivity of 1.890S/m.

**Fig. 15:**
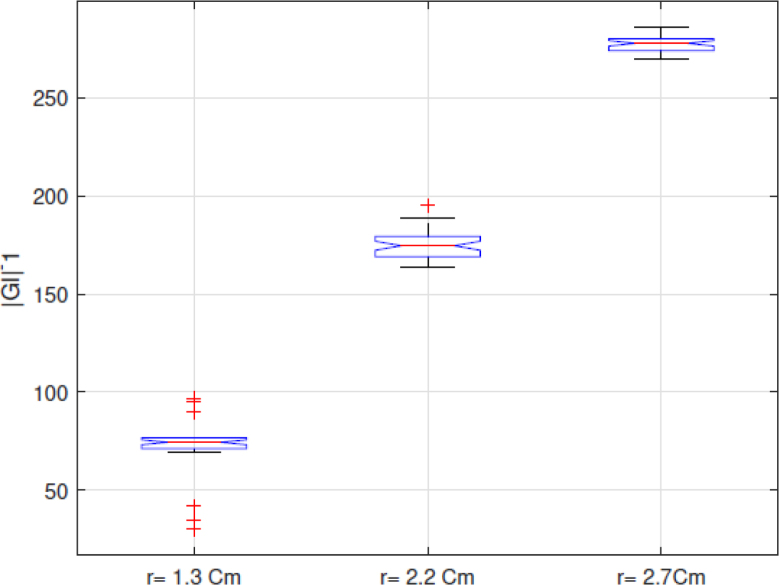
${\left|GI\right|}^{-1}$ for conductivity of 2.070S/m.

Because the ${\left|GI\right|}^{-1}$ index, which is the sum of the pixels of the EIT images in each frame and presents a positive correlation with the volume, as indicated in [25], is used in this work to analyze the performance of the implemented EIT-PSoC. The behavior of the ${\left|GI\right|}^{-1}$ index is graphed to support the volume estimation study. Fig. 13, Fig. 14, Fig. 15 show the results; evidencing that the volume of the cavity with saline solution is directly proportional to ${\left|GI\right|}^{-1}$, corroborating the results presented in [25].

**Fig. 16:**
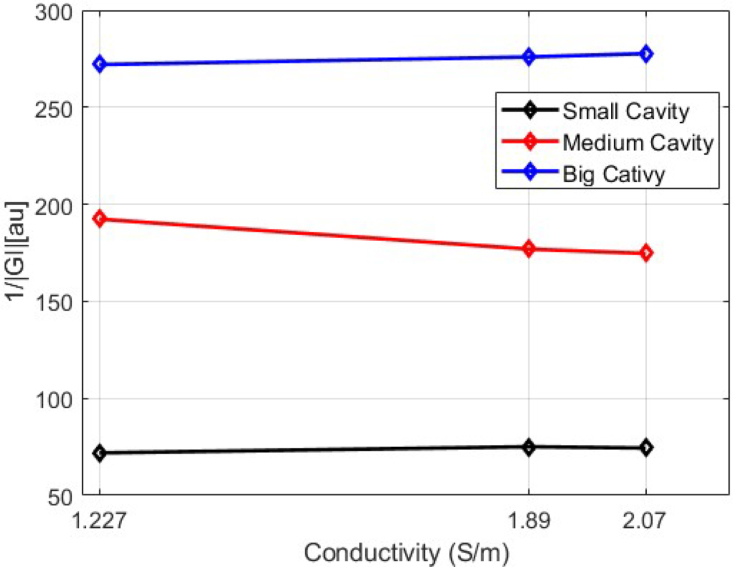
Trends in ${\left|GI\right|}^{-1}$ medians for agar phantoms.

Finally, Fig. 16 shows the trend of the medians of the data sets for each experiment with the agar phantoms, showing that the ${\left|GI\right|}^{-1}$ index and the designed EIT system would allow determining the difference in body fluid volume.

Comparing the results of EIT-PSoC system with other systems (Table 7), it can be seen that the system proposed in this work, presents the best frame frequency; also, the frequency range of operation and the SNR are characteristics to improve.

**Table 7:** Comparison of the portables EIT system [7].

	SNR (dB)	Frame rate (fps)	Frequency (Khz)
Miniaturized EIT	70	20	50
EIT SoC	56.3	20	10, 50, 100, 200
Microminiaturizing EIT	*	*	10–200
OXBACT-5 EIT	73.9	25	1–100
WMFEIT	70	70	1–1000
EIT-PSoC (Proposed system)	63.59	100	1–50

## CRediT authorship contribution statement

**Fausto Andrés Escobar:** Writing – original draft, Software, Investigation, Formal analysis, Data curation, Conceptualization. **Carlos Felipe Rengifo:** Writing – review & editing, Supervision, Methodology. **Víctor Hugo Mosquera:** Writing – review & editing, Writing – original draft, Supervision, Software, Methodology, Investigation.

## Declaration of competing interest

The authors declare no conflicts of interest related to the publication. No financial or personal relationships with other people or organizations have influenced or could be perceived to influence the content of this work.
